# Visual and Optical Absorbance Detection of Melamine in Milk by Melamine-Induced Aggregation of Gold Nanoparticles

**DOI:** 10.3390/nano11051142

**Published:** 2021-04-28

**Authors:** Shafiquzzaman Siddiquee, Suryani Saallah, Noor Aini Bohari, Gilbert Ringgit, Jumardi Roslan, Laila Naher, Nur Fatihah Hasan Nudin

**Affiliations:** 1 Biotechnology Research Institute, Universiti Malaysia Sabah, Jalan UMS, Kota Kinabalu 88400, Sabah, Malaysia; shafiqpab@ums.edu.my (S.S.); noorainibohari@gmail.com (N.A.B.); gilbertringgit@gmail.com (G.R.); 2 Faculty of Food Science and Nutrition, Universiti Malaysia Sabah, Jalan UMS, Kota Kinabalu 88400, Sabah, Malaysia; jumardi@ums.edu.my; 3 Faculty of Agro Based Industry, Universiti Malaysia Kelantan, Jeli 17600, Kelantan, Malaysia; lailanaher@umk.edu.my; 4 Faculty of Bioresources and Food Industry, Universiti Sultan Zainal Abidin, Besut 22200, Terengganu, Malaysia; fatihah@unisza.edu.my

**Keywords:** gold nanoparticles, melamine, optical absorbance, UV–Vis, infant formula

## Abstract

The present study reported a facile method for the determination of melamine in milk powder products based on the aggregation of reactant-free 5 nm gold nanoparticles (AuNPs). The strong electrostatic attraction between the positively charged exocyclic amine groups present in the melamine molecule and the negatively charged ions bound to the AuNPs induced aggregation of the AuNPs, resulting in visible color changes that could be seen with the naked eye and monitored by ultraviolet–visible (UV–Vis) absorbance spectra. The method shows high sensitivity with detection limits of 1 × 10^−9^ M for visual detection and 1 × 10^−11^ M for UV–Vis analysis, which is far below the safety limit of melamine ingestion in infant formula (1 ppm = 7.9 × 10^−6^ M) and the detection limit acquired by most AuNP-based melamine detection methods. Good recoveries were obtained over the range of 94.7–95.5% with a relative standard deviation of mean recovery (RSD) ranging from 1.40 to 5.81. The method provides a simple, feasible, fast and real-time detection of melamine adulterants in infant formula by the naked eye, without the aid of advanced instruments.

## 1. Introduction

Melamine is an organic base of trimer of cyanamide with a 1,3,5-triazine skeleton (C_3_H_6_N_6_), commonly used in the production of plastics, coatings, commercial filters, adhesives, among others [1,2,3]. As melamine contains a substantial amount of nitrogen (66% by mass), it was often adulterated into human and animal food to increase the protein content [2]. Excessive and continuous exposure to melamine have been linked to renal failure, kidney stones and associated deaths in infants and pets [4]. This alarming situation has created an urgent need for the continuous monitoring of melamine level in food products, especially in infant milk powder. A safety limit of melamine ingestion has been officially set, by the US FDA (Food and Drug Administration), at 2.5 ppm for adults and at 1 ppm for infant formula [5].

To date, various analytical techniques have been applied for the determination of melamine in milk products, which mainly rely on chromatographic [6,7], spectroscopic [8,9] and other separation methods, such as ELISA [10] and capillary electrophoresis [11]. These methods are well known for their high sensitivity and accuracy. Nevertheless, the complex and time-consuming sample preparation procedures, expensive instrumentation, and the requirement for high-skilled operators have impeded their application. Therefore, a simple and rapid method for on-site and real-time detection of melamine is highly intriguing and in great demand.

On the basis of their easy preparation and functionalization, exceptional biostability and distinct spectral properties, gold nanoparticles (AuNPs) have attracted great interest as a probe for melamine detection. Their unique size- and interparticle distance-dependent optical properties enable visual molecular recognition through color changes, observable by the naked eye [12,13,14]. However, most of the AuNP-based melamine detection methods that have been reported so far require complex modification of the AuNPs to enhance the sensitivity, thus limiting their application to some extent. The attempt to use citrate-capped AuNPs provides a simple means of AuNP modification, but with compromised detection sensitivity [15]. Chen et al. (2012) [16] reported that bare 8 nm AuNPs prepared by the borohydride reduction method can be used for detection of melamine. The bare AuNPs have an abundance of active surface gold atoms that enable the binding of melamine to AuNPs to occur via electrostatic interaction. The proposed method shows high sensitivity with a detection limit of 2.0 × 10^−7^ g·L^−1^, far lower than that of the citrate-capped and many other AuNP-based melamine detection methods, but suffers from time-consuming sample pre-treatment.

Herein, a simple method was proposed for the rapid detection of melamine using reactant-free 5 nm AuNPs stabilized in PBS as a probe. The mechanism of melamine-induced aggregation of AuNPs could enable naked eye observation, as well as optical absorbance detection with UV–Vis spectroscopy. We hypothesized that the use of standard colloidal AuNPs with a smaller size will provide more reliable, reproducible and sensitive melamine detection than the self-synthesized AuNPs. The effects of pH, interaction time, and temperature on the UV–Vis absorbance were investigated in order to find the optimum assay conditions. The developed method was then used to examine the presence of melamine in infant formula to validate its reliability in practical applications.

## 2. Materials and Methods

### 2.1. Reagents and Materials

Melamine, trichloroacetic acid, gold nanoparticles (AuNPs) with a 5 nm diameter suspended in 0.1 mM phosphate buffer (PB) solution, disodium hydrogen phosphate (Na_2_HPO_4_) and sodium dihydrogen phosphate (NaH_2_PO_4_) were purchased from Sigma Aldrich (St. Louis, MO, USA). The melamine was prepared by dissolving it with 10 mM PB solution (pH 7). All the solvents and reagents were of analytical reagent grade and were used without further purification. Milli-Q purified distilled water was used throughout the experiments. All the stock solutions were prepared daily with double-deionized water obtained from a Milli-Q water purification system. The infant formula was purchased from the local supermarket in Kota Kinabalu, Sabah, Malaysia.

### 2.2. Apparatus and Equipment

Absorbance spectra were recorded using a Lambda 35 UV–Vis spectrophotometer (Perkin Elmer, Norwalk, CT, USA) with 3.5 mL quartz cells at an absorbance ratio of A_650_/A_450_. A Hitachi S-3400N (Hitachi, Japan) scanning electron microscope (SEM) and Tecnai G2 Spirit Bio TWIN transmission electron microscope (TEM) (Hillsboro, OR, USA) imaging were used to observe the morphological properties of the AuNPs and melamine, and to confirm the aggregation of the AuNPs. All the experiments were conducted at room temperature conditions of 21 ± 2 °C.

### 2.3. Optimization of Assay Conditions

The performance of the developed melamine method is strongly influenced by the assay conditions, which include the pH, interaction time and binding temperature. Optimization of the assay conditions on the absorbance ratio in the presence of 9.6 × 10^−3^ M melamine were investigated in the range of pH 1 to 10, 1 to 10 min interaction time and 4 to 70 °C binding temperature. The pH of the solution was adjusted using either sodium hydroxide (1 M) or hydrochloric acid (1 M) solutions. The pH measurements were performed with pH-2700 Eutech Instruments (Postbus, Landsmeer, The Netherlands).

### 2.4. Visual and UV–Vis Detections of Melamine

The assay solution was prepared by mixing AuNPs with melamine at different concentrations in the range of 1 × 10^−10^ to × 10^−2^ M. The AuNPs (without melamine) were used as a control. The color changes were observed visually by the naked eye and recorded by a digital camera or by measuring the absorbance ratio with the UV–Vis spectrometer (Perkin Elmer, Norwalk, CT, USA) to determine the detection limit.

### 2.5. Pre-Treatment of Milk Sample

Infant formula was bought from a local supermarket in Kota Kinabalu, Sabah, Malaysia and pre-treated according to Li et al. (2010) [13], with slight modifications. Firstly, 1 g of milk powder sample was mixed with 2 mL of water in a centrifuge tube. The resulting milk suspension was shaken for 1 min, followed by the addition of 2 mL of trichloroacetic acid. The mixture was then sonicated for 10 min and centrifuged for 5 min at 3500 rpm. The supernatant was transferred into a new centrifuge tube and filtered with 0.22 µm filter paper and adjusted to pH 7.

### 2.6. Recovery Assay of Spiked Melamine in Milk Sample

The recovery assay was conducted by spiking 1 × 10^−5^ M and 1 × 10^−6^ M to the pre-treated sample. The spiked sample (500 µL) was then mixed with 500 µL of the prepared AuNPs and left to react for 7 min at room temperature. The absorption spectrum of the solution was then recorded by the UV–Vis spectrophotometer with an absorption ratio of (A_600_/A_520_) to obtain the relative standard deviation (RSD) and recovery value.

## 3. Results and Discussion

### 3.1. Morphological Characterization of AuNPs

**Figure 1:**
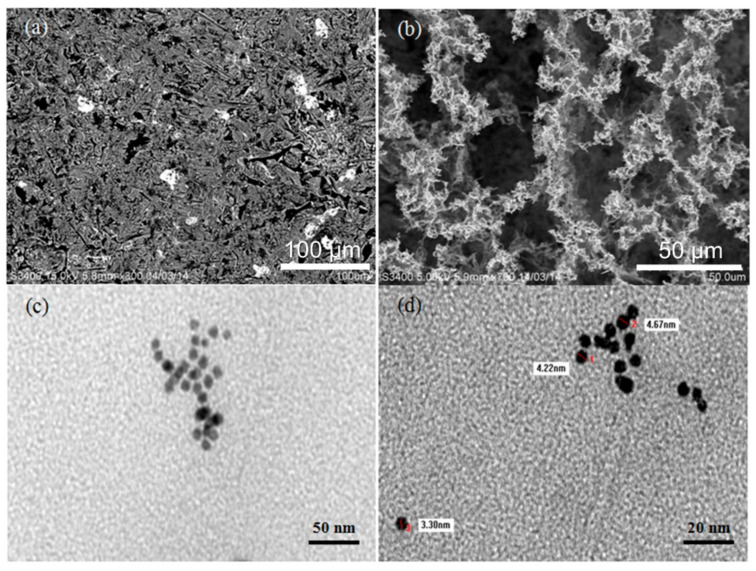
SEM of AuNPs (**a**), AuNPs/melamine (**b**), and TEM of AuNPs (**c**,**d**).

The morphological properties of the AuNPs and AuNPs/melamine were characterized by SEM and TEM, and the results are presented in Figure 1. In contrast with the homogeneously distributed AuNPs (Figure 1a), clustering was observed for the AuNPs/melamine where the particles were aggregated and formed interconnected networks among each other (Figure 1b). The melamine-induced aggregation of AuNPs was further confirmed by the TEM images in Figure 1c,d. The AuNPs are spherical in shape with a size of approximately 3–5 nm for the individual nanoparticles, while the size of the aggregated AuNPs is about 50 nm, which agrees well with the TEM result reported by Kumar et al. (2014) [15].

### 3.2. Effects of Addition of Melamine to the AuNPs

**Figure 2:**
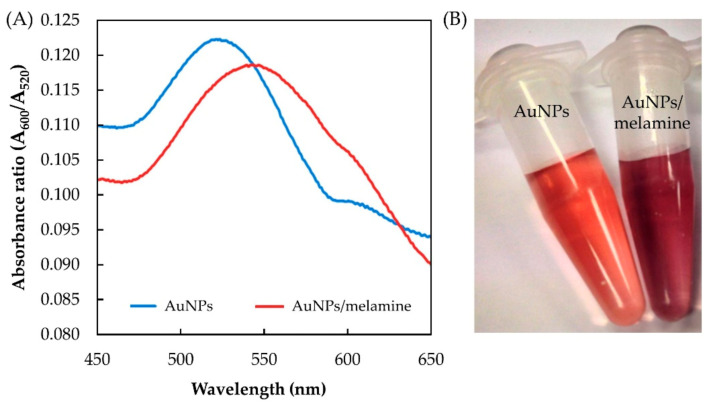
Absorbance spectra (**A**) and the color changes of AuNPs and AuNPs/melamine (**B**).

The absorption peak of AuNPs is closely related to the space between the nanoparticles. Obvious shifting of the absorption peaks will occur when the state of the AuNPs changes from dispersive to aggregated. The AuNPs colloidal solution shows a typical surface plasmon resonance (SPR) band in the proximity of 520 nm (Figure 2A), indicating the AuNPs are well dispersed [17]. The addition of melamine to the AuNPs colloid causes the SPR peak to slightly reduce and shift to a higher wavelength, associated with the aggregation of the AuNPs (Figure 2B) [16].

**Figure 3:**
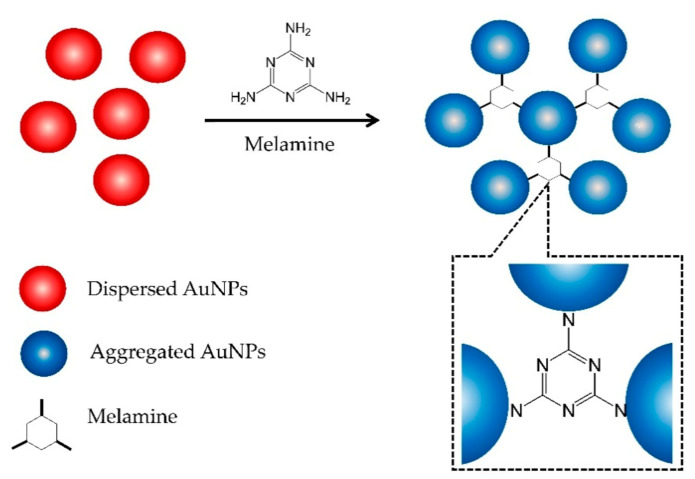
Schematic illustration of the melamine-induced AuNPs aggregation mechanism.

Figure 3 shows the mechanism of AuNPs aggregation in the presence of melamine. Initially, the AuNPs are in the form of a stable colloidal solution due to the presence of an electrostatic layer that keeps them separated. Strong electrostatic attraction, created by the positively charged exocyclic amino groups (–NH_2_) in the melamine and the negatively charged AuCl_4_^−^/AuCl_2_^−^ ions bound on the surface of the AuNPs, reduces the stability of the AuNPs, leading to aggregation [15,16]. This aggregation caused the appearance of new absorption peak at 600 nm (Figure 2A), ascribed to the dipole–dipole interaction between plasmons of adjacent particles in the aggregates [15].

### 3.3. Optimization of Assay Conditions

#### 3.3.1. Effect of pH

**Figure 4:**
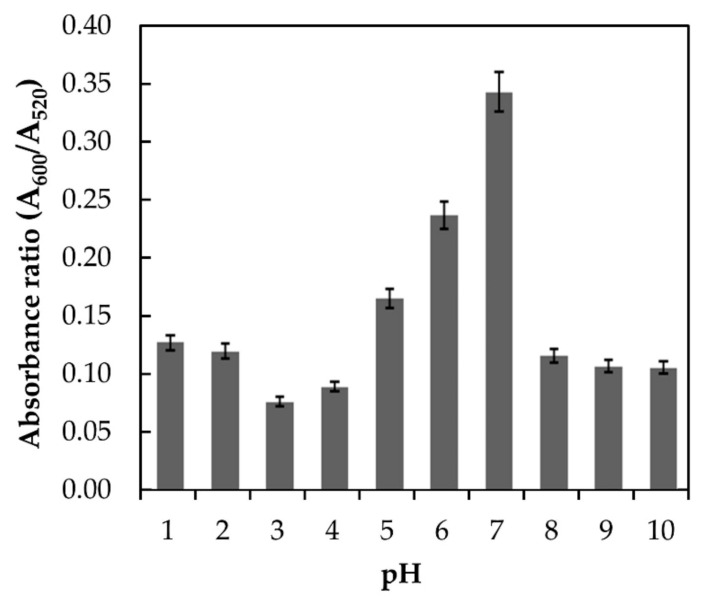
Absorbance of AuNPs in the presence of 9.6 × 10^−3^ M melamine at different pH with absorbance ratios of (A_600_/A_520_).

Melamine is a weak base with a pKa of 5.05. Therefore, the form of melamine in an aqueous solution will largely be affected by the media pH [18]. The interaction of melamine and AuNPs at different pH conditions will influence the colloidal stability of the AuNPs, which eventually affects the spectroscopic signature of the surface plasmon resonance wavelength [13,19]. In this study, the effect of pH was investigated in the range of 1 to 10, and the responses in terms of absorbance ratios (A_600_/A_520_) were plotted, as shown in Figure 4.

The highest absorbance ratio was obtained at neutral pH (pH 7.0). In acidic and basic media, a lower absorbance ratio was observed. The reason behind the lower absorbance ratio in acidic and basic media could be related to the fact that melamine was hydrolyzed and became unstable due to the loss of its amino groups. These amino groups were gradually replaced by hydroxyl groups and finally transformed melamine into cyanuric acid which inhibits the AuNPs aggregation. It is also worth to note that at relatively low (acidic) and high pH (basic), partial aggregation of the AuNPs may occur which hindered the interaction between melamine and AuNPs. A similar trend was also reported by Li et al. (2010) [13] and Su et al. (2011) [19] where the highest absorbance ratio for melamine and AuNPs interaction was obtained at pH 7. Therefore, pH 7 was selected as the optimum media pH.

#### 3.3.2. Effect of Interaction Time

**Figure 5:**
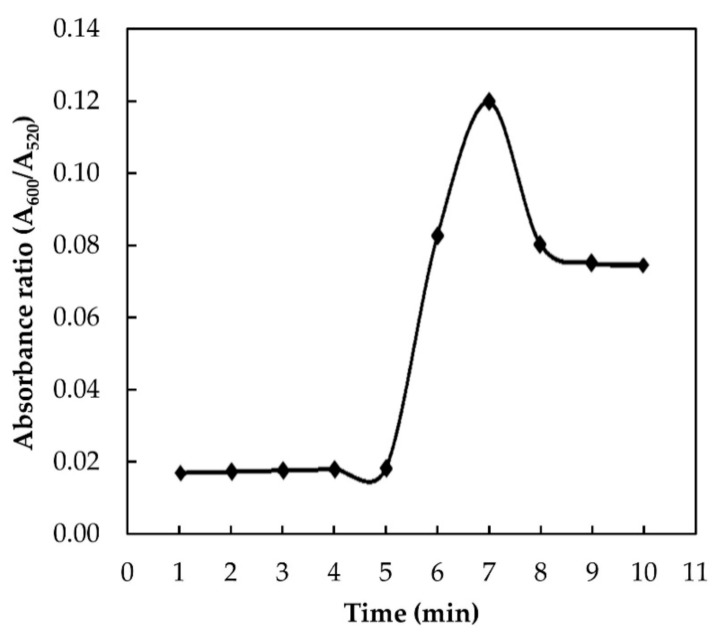
Effect of interaction time on absorbance ratio (A_600_/A_520_) of AuNPs in the presence of 9.6 × 10^−3^ M melamine.

The interaction time plays an important role in the determination of the aggregation kinetics [16]. The optimum time of the proposed assay was examined by adding 9.6 × 10^−3^ M of melamine into the AuNPs colloidal solution and leaving the sample to react at different time intervals, ranging from 1 to 10 min. The absorbance ratio against time was plotted and presented in Figure 5. For the first 5 min, no change in the absorbance ratio was observed. This indicates that no interaction occurs between melamine and AuNPs at these points. Melamine–AuNPs reaction started to occur after 5 min and reach its maximum absorption value at 7 min. After that, the reaction plateaued, indicating that the aggregation of AuNPs with melamine was entirely completed within 7 min. Therefore, the optimum reaction time was selected as 7 min for further analysis.

#### 3.3.3. Effect of Temperature

**Figure 6:**
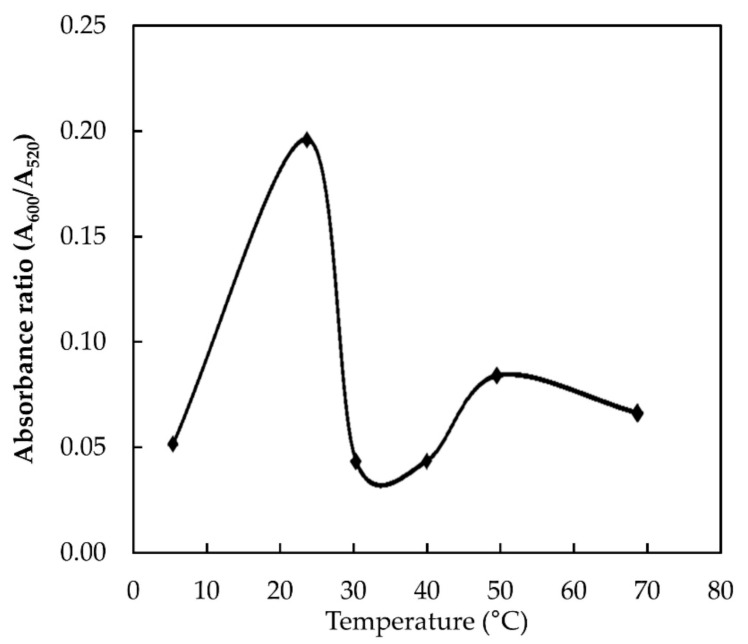
Interaction of AuNPs with 9.6 × 10^−3^ M melamine at different temperatures.

Besides pH and reaction time, temperature is also one of the key factors that influences the binding ability of melamine and AuNPs [13]. To determine the effect of temperature on the absorbance ratio, the temperature was varied from 4 to 70 °C and the results are shown in Figure 6. The highest absorbance ratio was achieved at the room temperature condition, indicating the optimum binding between melamine and AuNPs occur at this temperature. However, further increase in the temperature reduces the absorbance ratio drastically. A similar observation was also reported by Li et al. (2010) [13]. This could be due to the instability of AuNPs at high temperatures, which causes self-aggregation of the AuNPs and reduces their interaction with melamine [13,16]. Therefore, 20 °C was chosen as an optimal temperature.

### 3.4. Detection of Melamine

**Figure 7:**
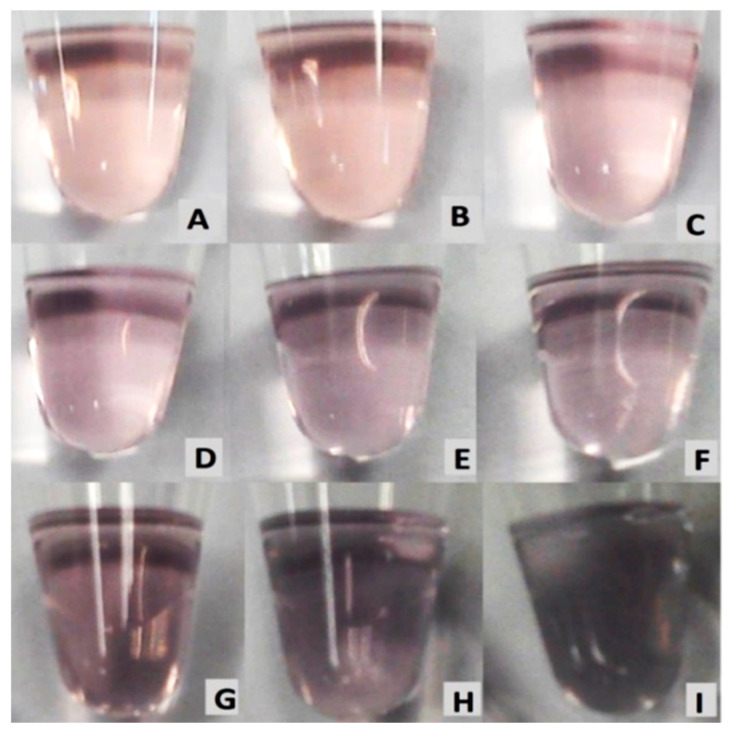
Visual color change of (**A**) AuNPs, and AuNPs in the presence of the following different concentrations of melamine: (**B**) 1 × 10^−9^ M, (**C**) 1 × 10^−8^ M, (**D**) 1 × 10^−7^ M, (**E**) 1 × 10^−6^ M, (**F**) 1 × 10^−5^ M, (**G**) 1 × 10^−4^ M, (**H**) 1 × 10^−3^ M, and (**I**) 1 × 10^−2^ M.

The detection of melamine is conducted by the addition of melamine with a concentration ranging from 1 × 10^−10^ to × 10^−2^ M. AuNPs (without melamine) were used as a control. The color changes were observed visually by naked eye and recorded by a digital camera, and by measuring the absorbance ratio with UV–Vis spectrophotometer. As shown in Figure 7, gradual color changes from wine-red to dark purple can be clearly observed and associated with the increasing concentrations of melamine. This observation confirmed the aggregation of AuNPs by melamine, resulting in visible color changes. The shade of color in Figure 7A of AuNPs and in Figure 7B of AuNPs/melamine at a concentration of 1 × 10^−9^ M was very close. Therefore, the visual color detection limit was determined to be 1 × 10^−9^ M.

**Figure 8:**
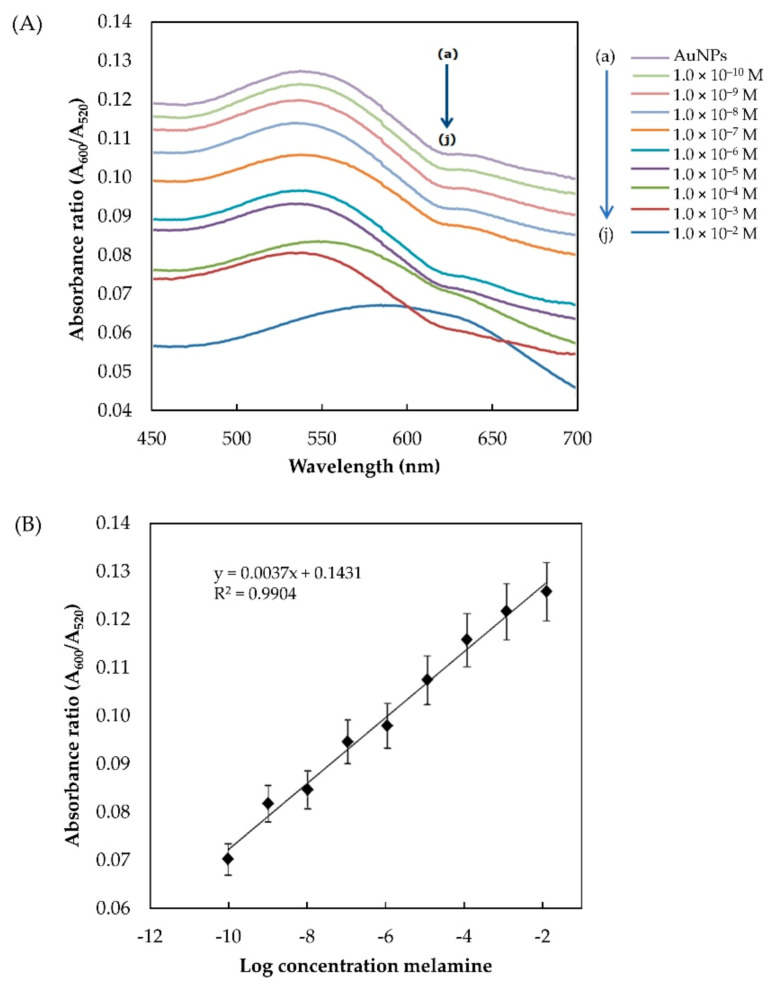
(**A**) Absorption spectra of the AuNPs generated in the presence of different concentrations of melamine (a–j: control, 1 × 10^−10^, 1 × 10^−9^, 1 × 10^−8^, 1 × 10^−7^, 1 × 10^−6^, 1 × 10^−5^, 1 × 10^−4^, 1 × 10^−3^, and 1 × 10^−2^ M); (**B**) plot of A_600_/A_520_ against the log of melamine concentrations (*n* = 5).

The absorbance ratio A_600_/A_520_ was calculated based on the spectra obtained from UV–Vis analysis of the control (AuNPs) and AuNPs/melamine. As shown in Figure 8A, the addition of 1 × 10^−10^ to × 10^−2^ M melamine to the AuNPs colloidal solution resulted in a gradual reduction in the absorbance ratio. A linear regression equation for the calibration plot was calculated to be (A_600_/A_520_) = 0.0073 (M) + 0.1431 with a good linear correlation (R^2^ = 0.9904) (Figure 8B). The UV–Vis detection limit is at 1 × 10^−11^ M (0.00125 ppb) (*n* = 5). Considering the safety limits of melamine ingestion (2.5 ppm in the USA and EU; 1 ppm in infant formula in China), this method definitely meets the requirements of routine detection. Furthermore, the developed method is much simpler than the existing methods without the need for complicated AuNP modification and sample pre-treatment. The detection limit achieved here is 0.00125 ppb, well below the safety limit of melamine ingestion (1 ppm). The use of 5-nm commercial AuNPs as a probe is highly reproducible and sensitive for the rapid screening of infant formula samples in a quick and simple manner. Table 1 shows the comparison between the detection limit achieved in the present study and other methods reported in the literature for melamine detection using AuNPs as a probe.

### 3.5. Analytical Application

The developed method was used to examine the presence of melamine in infant formula to validate its reliability in practical applications [21,22]. According to Chen et al. (2012) [16], the detection of melamine is often affected by the interference of macromolecular compounds such as protein which is present in milk. Therefore, in order to reduce the matrix effect, sample pre-treatment is necessary [13,16,23]. In this study, the infant formula was pre-treated with trichloroacetic acid using the method in Section 2.5. The sample was then spiked with melamine at a concentration of 1 × 10^−5^ M and 1 × 10^−6^ M, which represent the melamine concentration within the legislative value of 1 to 2.5 ppm. Based on the results presented in Table 2, the recovery rates were found to be 95.5% for 1 × 10^−5^ M and 94.7% for 1 × 10^−6^ M, with a RSD of 5.77% and 1.39%, respectively. According to Kumar et al. (2014) [15], a recovery rate of ~95% and a low RSD indicate high accuracy and precision of the method. Therefore, the findings of the present study suggested that the proposed method is positively applicable for melamine determination in milk powder samples.

## 4. Conclusions

A simple and rapid method was developed based on bare AuNPs as a probe for visual and optical detections of melamine by the naked eye and a UV–Vis spectrometer. The method is proven to be highly sensitive with a detection limit far below the legislative safety limits of melamine ingestion. The presented method was also successfully applied to detect melamine in infant formula with good recoveries (~95%) and a low RSD. Findings of the present study suggest that the bare AuNP-based melamine detection method is promising for the rapid screening and detection of melamine adulterants in infant formula and other milk samples without the aid of advanced instruments. The data obtained from this study could be useful for the development of a portable melamine test kit.

## Figures and Tables

**Table 1:** Comparison of detection limits obtained in the present study with some established methods using AuNPs as a probe.

Type of AuNPs	LOD (M)	Reference
Unmodified	1.59 × 10^−9^	[20]
Unmodified	3.17 × 10^−6^	[13]
Gold (during formation)	8.00 × 10^−9^	[12]
Gold (non-aggregation)	8.00 × 10^−10^	[14]
3-mercapto-1-propanesulfonate modified	8.00 × 10^−9^	[19]
Bare AuNPs (8 nm)	3.96 × 10^−7^ (visual) 1.56 × 10^−9^ (UV–VIS)	[16]
Synthetic AuNPs (5 nm)	1.00 × 10^−9^ (visual) 1.00 × 10^−11^ (UV–VIS)	This work

**Table 2:** Determination of melamine in real milk samples spiked with different concentrations of melamine.

Sample	Melamine Added ( 10^−5^ M)	Detected (10^−5^ M)	RSD (%)	Recovery (%)
1	1	0.96	5.81	95.5
2	0.1	0.094	1.40	94.7

## Data Availability

Not applicable.

## References

[1] Mecker L.C., Tyner K.M., Kauffman J.F., Arzhantsev S., Mans D.J., Gryniewicz-Ruzicka C.M. (2012). Selective melamine detection in multiple sample matrices with a portable Raman instrument using surface enhanced Raman spectroscopy-active gold nanoparticles. Anal. Chim. Acta.

[2] Xu X.M., Ren Y.P., Zhu Y., Cai Z.X., Han J.L., Huang B.F., Zhua Y. (2009). Direct determination of the melamine in dairy products by gas chromatography/mass spectrometry with coupled column separation. Anal. Chim. Acta.

[3] Mauer L.J., Chernyshova A.A., Hiatt A., Deering A., Davis R. (2009). Melamine detection in infant formula powder using near-and mid-infrared spectroscopy. J. Agric. Food Chem..

[4] Gossner C.M., Shlundt J., Embarek P.B., Hird S., Wong L.F.D., Javier J., Beltran O., Teoh K.N., Tristscher A. (2009). The melamine incident: Implication for international food and feed safety. Environ. Health Perspect..

[5] FDA (2009). Melamine Pet Food Recall in 2007. http://www.fda.gov/oc/opacom/hottopics/petfood.html.

[6] Sun H.W., Wang L.X., Ai L.F., Liang S.X., Wu H. (2010). A sensitive and validated method for determination of melamine residue in liquid milk by reversed phase high-performance liquid chromatography with solid-phase extraction. Food Control.

[7] Venkatasami G., Sowa J.R. (2010). A rapid, acetonitrile-free, HPLC method for determination of melamine in infant formula. Anal. Chim. Acta.

[8] Jawaid S., Talpur F.N., Hassan I.A. (2014). Quick determination of melamine in infant powder and liquid milk by Fourier transform infrared spectroscopy. Anal. Methods.

[9] Nieuwoudt M., Holroyd S., McGoverin C., Simpson M., Williams D. (2016). Raman spectroscopy as an effective screening method for detecting adulteration of milk with small nitrogen-rich molecules and sucrose. J. Dairy Sci..

[10] Sun F., Liu L., Hua K., Chuanlai K. (2013). Development of ELISA for melamine detection in milk powder. Food Agric. Immunol..

[11] Kong Y., Wei C., Hou Z., Wang Z., Yuan J., Yu J., Zhao Y., Tang Y., Gao M. (2014). Stacking and analysis of melamine in milk products with acetonitrile-salt stacking technique in capillary electrophoresis. J. Anal. Methods Chem..

[12] Wu Z., Zhao H., Xue Y., Cao Q., Yang J., He Y., Li X., Yuan Z. (2011). Colorimetric detection of melamine during the formation of AuNPs. Biosens. Bioelectron..

[13] Li L., Li B., Cheng D., Mao L. (2010). Visual detection of melamine in raw milk using gold nanoparticles as colorimetric probe. Food Chem..

[14] Cao Q., Zhao H., He Y., Li X., Zeng L., Ding N., Wang J., Yang J., Wang G. (2010). Hydrogen-bonding induced colorimetric detection of melamine by nonaggregation based AuNPs as a probe. Biosens. Bioelectron..

[15] Kumar N., Seth R., Kumar H. (2014). Colorimetric detection of melamine in milk by citrate-stabilized gold nanoparticles. Anal. Biochem..

[16] Chen W., Deng H.H., Hong L., Wu Z.Q., Wang S., Liu A.L., Lin X.H., Xia X.H. (2012). Bare gold nanoparticles as facile and sensitive colorimetric probe for melamine detection. Analyst.

[17] Liu S., Kannegulla A., Kong X., Sun R., Liu Y., Wang R., Yu Q., Wang A.X. (2020). Simultaneous colorimetric and surface-enhanced Raman scattering detection of melamine from milk. Spectrochim. Acta A.

[18] El-sheikh A.H., Al-degs Yahya S., Abu-Wardeh A.H., Al-ghouti Mohammad A. (2017). Quantification of Melamine in Milk and Dairy Products by Liquid Chromatography after a Simple Sample Clean-Up Procedure. J. Food Process. Preserv..

[19] Su H., Fan H., Ai S., Wu N., Fan H., Bian P., Liu J. (2011). Selective determination of melamine in milk samples using 3-mercapto-1-propanesufonate-modified gold nanoparticles as colorimetric probe. Talanta.

[20] Guan H., Yu J., Chi D. (2013). Label-free colorimetric sensing of melamine based on chitosan-stabilized gold nanoparticles probes. Food Control.

[21] Song J., Wu F., Wan Y., Ma L.-H. (2014). Visual test for melamine using silver nanoparticles modified with chromotropic acid. Microchim. Acta.

[22] Chang K., Wang S., Zhang H., Guo Q., Hu X., Lin Z., Sun H., Jiang M., Hu J. (2017). Colorimetric detection of melamine in milk by using gold nanoparticles-based LSPR via optical fibers. PLoS ONE.

[23] Huang H., Li L., Zhou G., Liu Z., Ma Q., Feng Y., Zeng G., Tinnefeld P., He Z. (2011). Visual detection of melamine in milk samples based on label-free and labelled gold nanoparticles. Talanta.

